# Revealing the specific regulations of nitric oxide on the postharvest ripening and senescence of bitter melon fruit

**DOI:** 10.1007/s42994-023-00110-y

**Published:** 2024-03-21

**Authors:** Hongwei Wang, Ling Li, Lili Ma, Alisdair R. Fernie, Anzhen Fu, Chunmei Bai, Zhaoze Sang, Susu Guo, Fan Zhang, Qing Wang, Yanyan Zheng, Jinhua Zuo

**Affiliations:** 1 Key Laboratory of Vegetable Postharvest Processing, Ministry of Agriculture, Beijing Key Laboratory of Fruits and Vegetable Storage and Processing, Key Laboratory of Biology and Genetic Improvement of Horticultural Crops (North China) of Ministry of Agriculture, Key Laboratory of Urban Agriculture (North) of Ministry of Agriculture, Beijing Vegetable Research Center, Institute of Agro-Products Processing and Food Nutrition, Beijing Academy of Agriculture and Forestry Sciences, Beijing, 100097 China; 2 College of Food Science and Biotechnology, Tianjin Agricultural University, Tianjin, 300392 China; 3 Max Planck Institute of Molecular Plant Physiology, 14476 Potsdam Golm, Germany; 4 Institute of Food Science and Technology, Chinese Academy of Agricultural Sciences, Beijing, China

**Keywords:** Bitter melon, NO treatment, Postharvest, Ripening and senescence

## Abstract

Bitter melon fruit is susceptible to yellowing, softening, and rotting under room-temperature storage conditions, resulting in reduced commercial value. Nitric oxide (NO) is an important signaling molecule and plays a crucial role in regulating the fruit postharvest quality. In this study, we investigated the effects of NO treatment on changes in sensory and firmness of bitter melon fruit during postharvest storage. Moreover, transcriptomic, metabolomic, and proteomic analyses were performed to elucidate the regulatory mechanisms through which NO treatment delays the ripening and senescence of bitter melon fruit. Our results show that differentially expressed genes (DEGs) were involved in fruit texture (*CSLE*, *β-Gal*, and *PME*), plant hormone signal transduction (*ACS*, *JAR4*, and *AUX28*), and fruit flavor and aroma (*SUS2*, *LOX*, and *GDH2*). In addition, proteins differentially abundant were associated with fruit texture (PLY, PME, and PGA) and plant hormone signal transduction (PBL15, JAR1, and PYL9). Moreover, NO significantly increased the abundance of key enzymes involved in the phenylpropanoid biosynthetic pathway, thus enhancing the disease resistance and alleviating softening of bitter melon fruit. Finally, differential metabolites mainly included phenolic acids, terpenoids, and flavonoids. These results provide a theoretical basis for further studies on the physiological changes associated with postharvest ripening and senescence of bitter melon fruit.

**Supplementary Information:**

The online version contains supplementary material available at 10.1007/s42994-023-00110-y.

## Introduction

Bitter melon fruit (*Momordica charantia* L.) is a cucurbit family crop species that grows in tropical, subtropical, and temperate regions (Dimitrovski et al. 2020). Bitter melon fruit is refreshing and fragrant; has high nutritional value; and is rich in antioxidant substances, minerals, vitamins, and other nutritionally important trace elements (Barua et al. 2020). In medical research, bitter melon fruit has been shown to contain unique medicinal compounds, such as aromatic glycosides, flavor-related glycosides, and other alkaloids, which are thought to possess potent bioactivities against cardiovascular diabetes and cardiovascular and other diseases (Choudhary et al. 2022). Therefore, bitter melon fruits are popular among people all over the world for its high edibility and medicinal value (Devi et al. 2019). However, bitter melon fruit has a short shelf-life at room temperature, as yellowing of the skin, softening of the tissue, and loss of bitterness occur (Prajapati et al. 2021a).

Low-temperature storage, which slows the metabolic activity of bitter melon, is a practical and convenient way to extend storage time (Lin et al. 2020). Wang et al. (2007) found that bitter melon stored at 2 °C for 7 days still exhibited high levels of vitamin C, high contents of soluble protein, high microbiological safety, and high edible value. To date, several postharvest studies have been conducted to investigate the storage period of bitter melon fruit; these have included applications such as edible coatings or treatment with plant extracts to create film-like materials, which were shown to extend the storage period of bitter melon fruit by more than 7 days, thereby significantly improve shelf-life. A recent study showed that 1-MCP fumigation inhibited the yellowing of bitter melon fruit, delayed the process of fruit softening, and effectively extended the storage period and shelf-life of the fruit, which indicated that 1-MCP has a positive effect on the preservation of bitter melon fruit freshness (Han et al. 2015). In addition, UV-C, ethanol, and melatonin treatments have been shown to maintain the quality and shelf-life of bitter melon fruit (Prajapati et al. 2021a; b). There are, however, the effects of these methods on delaying the yellowing and softening of bitter melon is not significant or difficult to popularize in production.

Nitric oxide (NO) is an important redox signaling molecule in plants and plays an important role in regulating the physiology and quality of fruit and vegetables in terms of ripening, aging, and yellowing, as well as susceptibility to both disease and chilling injury (Buet et al. 2021). The application of NO at certain concentrations can delay the postharvest ripening process and improve the quality of fruit and vegetables after harvest (Yang and Liu 2017). Indeed, NO is commonly used to prolong the shelf-life of jujube (Zhao et al. 2019), apple (Steffens et al. 2021), and mullein. Li et al. (2020) treated postharvest peach fruit with 15 μmol/L NO solution, which not only enhanced the activities of defense enzymes, including polyphenol oxidase and peroxidase (POD), but also enhanced the disease resistance of the peach fruit. These facts notwithstanding, little information is available concerning the regulatory mechanisms of NO treatment on ripening of bitter melon fruit. In this study, transcriptomic, metabolomic, and proteomic analyses were used to reveal the regulatory mechanisms of NO treatment, at the molecular level, with regard to the physiological changes and delayed storage period of bitter melon fruit.

## Results

### Sensory score and firmness during storage

**Fig. 1:**
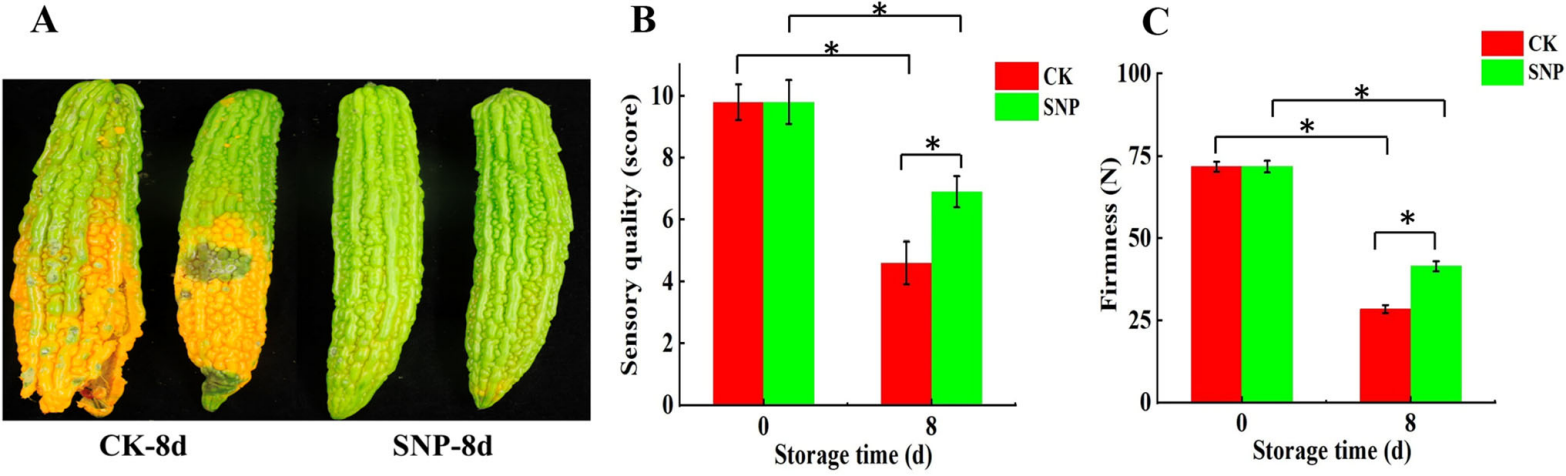
Changes in bitter melon fruit during storage after sodium nitroprusside (SNP) treatment. **A** Appearance. **B** Sensory quality. **C** Firmness. Asterisks indicate significant (*P* < 0.05) differences between means

The appearances of the controls and sodium nitroprusside (SNP)-treated bitter melon fruits are shown in Fig. 1A. Across the entire storage period, the sensory score of bitter melon fruit showed a downward trend. However, after storage for 8 days, the value (6.9) of the treated fruit was significantly higher than that of the untreated fruit (4.6) (Fig. 1B). The trend in fruit firmness change was consistent with that for sensory firmness. Similarly, the treated fruit maintained a higher firmness than did the untreated fruit (Fig. 1C). Taken together, these results showed that, compared with the control treatment, the SNP treatment delayed the ripening and senescence of fruit and maintained higher sensory quality and hardness of bitter melon.

### Effect of SNP treatment on gene expression in bitter melon fruit

**Fig. 2:**
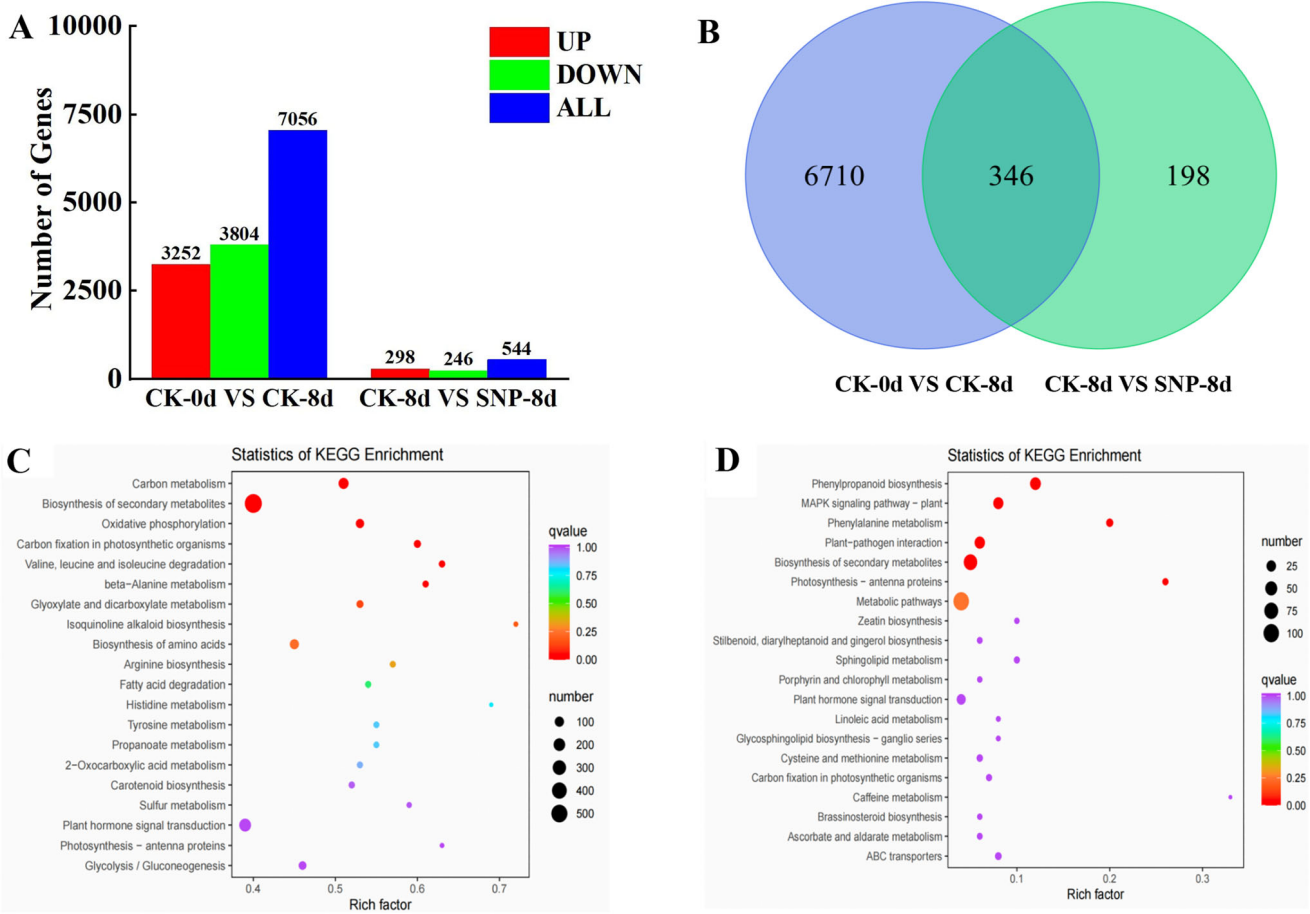
Number of differentially expressed mRNAs and results of a KEGG analyses. **A** Amounts of increased and decreased differentially expressed mRNAs in the CK-0d vs. CK-8d and CK-8d vs. SNP-8d comparison groups. **B** Number of differentially expressed mRNAs detected at the same time in the CK-0d vs. CK-8d and CK-8d vs. SNP-8d comparison groups. **C** Enriched KEGG pathways of genes differentially expressed in the CK-0d vs. CK-8d comparison group. **D** Enriched KEGG pathways of genes differentially expressed in the CK-8d vs. SNP-8d comparison group

A total of 7056 mRNAs were differentially expressed in the CK-0d versus CK-8d comparison group; namely, 3252 genes were upregulated, and 3804 genes were downregulated (Fig. 2A). These transcripts were mainly related to the biosynthesis of secondary metabolites, carbon metabolism, and amino acid biosynthesis pathways (Fig. 2C). SNP treatment affected the expression of 544 different mRNAs, with 246 being upregulated, and 298 undergoing downregulation (Fig. 2B). The related genes were significantly enriched in several metabolic pathways, including those involved in the biosynthesis of secondary metabolites, plant–pathogen interactions, and phenylpropanoid biosynthesis, as well as the MAPK signaling pathway-plant (Fig. 2D).

A total of 346 common differentially expressed mRNAs related to fruit ripening were detected in the CK-0d vs. CK-8d and CK-8d vs. SNP-8d comparison groups; these mRNAs corresponded to several important structural genes involved in fruit texture changes, plant hormone synthesis and signal transduction, and disease resistance, fruit color, and fruit flavor and aroma (Supplementary Appendix 1).

#### Genes involved in the texture of bitter melon fruit

The change in fruit texture seriously affects the storage, transportation, and commercial value of postharvest fruits. The softening of fruit is mainly caused by the degradation of pectin in the intercellular layer, which involves *polygalacturonase* (*PG*), *beta-galactosidase* (*BGAL*), *pectin methylesterase* (*PME*), and other enzymes (Yang et al. 2018). SNP treatment delayed the decrease in firmness of bitter melon fruit during storage. A total of 26 differentially expressed mRNAs related to fruit texture were identified in the CK-0d vs. CK-8d comparison group, of which 13 mRNAs increased and 13 mRNAs decreased. As shown in Table 1, some DEGs related to the cell wall, including *polygalacturonase 1 beta-like protein 3* (*PGL3*), *expansin-like A2* (*EXLA2*), *beta-galactosidase 8* (*BGAL8*), *callose synthase 5* (*CALS5*), *cellulose synthase A catalytic subunit 3* (*CESA3*)*,* and *cellulose synthase-like protein H1* (*CSLH1*), were upregulated, while *phenylalanine ammonia-lyase* (*PAL*)*, pectinesterase-like* (*PE1*), and *cellulose synthase-like protein E1* (*CSLE1*) were downregulated (Table 1).

**Table 1:** Differentially expressed mRNAs involved in fruit texture regulation during fruit ripening and senescence

ID	Nr annotation	Regulated	log2FC	
			CK0 vs CK8	CK8vs SNP8
LOC111025560	*Polygalacturonase 1 beta protein 3(PGL3)*	Down	3.03	− 1.89
LOC111007556	*Expansin A2 (EXLA2)*	Down	2.00	− 2.21
LOC111023780	*Beta-galactosidase 8 (BGAL8)*	Down	1.32	− 3.23
LOC111018891	*Callose synthase 5 (CALS5)*	Down	2.71	− 1.22
LOC111016610	*Cellulose synthase A catalytic subunit 3 (CESA3)*	Down	1.47	− 1.19
LOC111011340	*Cellulose synthase protein H1 (CSLH1)*	Up	1.93	1.06
LOC111020634	*Pectinesterase-like (PE1)*	Up	− 4.89	2.39
LOC111010435	*Cellulose synthase (CSLE1)*	Up	− 2.73	2.28
LOC111005294	*Cellulose synthase A catalytic subunit 2 (CESA2)*	Up	− 1.26	1.56

Eleven mRNAs related to bitter melon fruit texture were differentially expressed in the SNP-treated group compared with the control group; five of these mRNAs increased in expression, while six decreased. These differentially expressed mRNAs included *CESA3*, *expansin-like A2* (*EXLA2*), *CSLE1*, *cellulose synthase-like protein E6* (*CSLE6*), *CSLH1*, *pectinestrase 2* (*PE2*), *CESA3*, *CALS5*, *PE1, BGAL8*, and *PGL3* (Supplementary Appendix 2). The results further showed that the expression of the *PGL3* and *BGAL8* genes was significantly lower in the SNP treatment group than in the control group. After SNP treatment, the expression of *CALS5, CESA3,* and *expansin-like A2* (*EXLA2*) was significantly inhibited. We also identified genes that were independently regulated by SNP during the ripening and senescence process of bitter melon fruits. We identified seven fruit texture related genes, mainly including *expansin-like*, *cellulose synthase-like protein*, *beta-galactosidase-like,* and *polygalactosidase QRT3-like*. The differential expression of these mRNAs may be related to the changes in fruit texture, indicating that SNP treatment had potential effects on the soft process of bitter melon fruit during storage.

#### Genes involved in plant hormone synthesis and hormone perception

As is well known, plant hormones are crucial signal compounds for plants to regulate growth, development, and response to environmental stress. The genes encoding ethylene biosynthesis and signal transduction play a regulatory role during the fruit ripening process, including *1-aminocyclopane-1-carboxylate synthesis* (*ACS*), *1-aminocyclopane-1-carboxylate oxidation* (*ACO*), and *ethylene responsive factor* (*ERF*) (Zhang et al. 2018). Fifteen mRNAs related to plant hormones and their perception and signal transduction were differentially expressed in the CK-8d vs. SNP-8d comparison group. Eleven genes were upregulated after the SNP treatment compared with the control treatment, and four genes were downregulated. These differentially expressed mRNAs included *auxin response protein IAA8-like* (*IAA8*), *3-epi-6-deoxocatharsterone 23-monooxygenase* (*CYB90D1*), *EIN3-binding F-box protein 1* (*EBF2*), *1-aminocyclopropane-1-carboxylate synthesis* (*ACS*), *transcription factor bHLH128* (*bHLH 128*), *jasmonic acid-amido synthetase JAR1-like* (*JAR4*), and *abscisic acid receptor PYL9* (*PYL9*) (Supplementary Appendix 3).

**Fig. 3:**
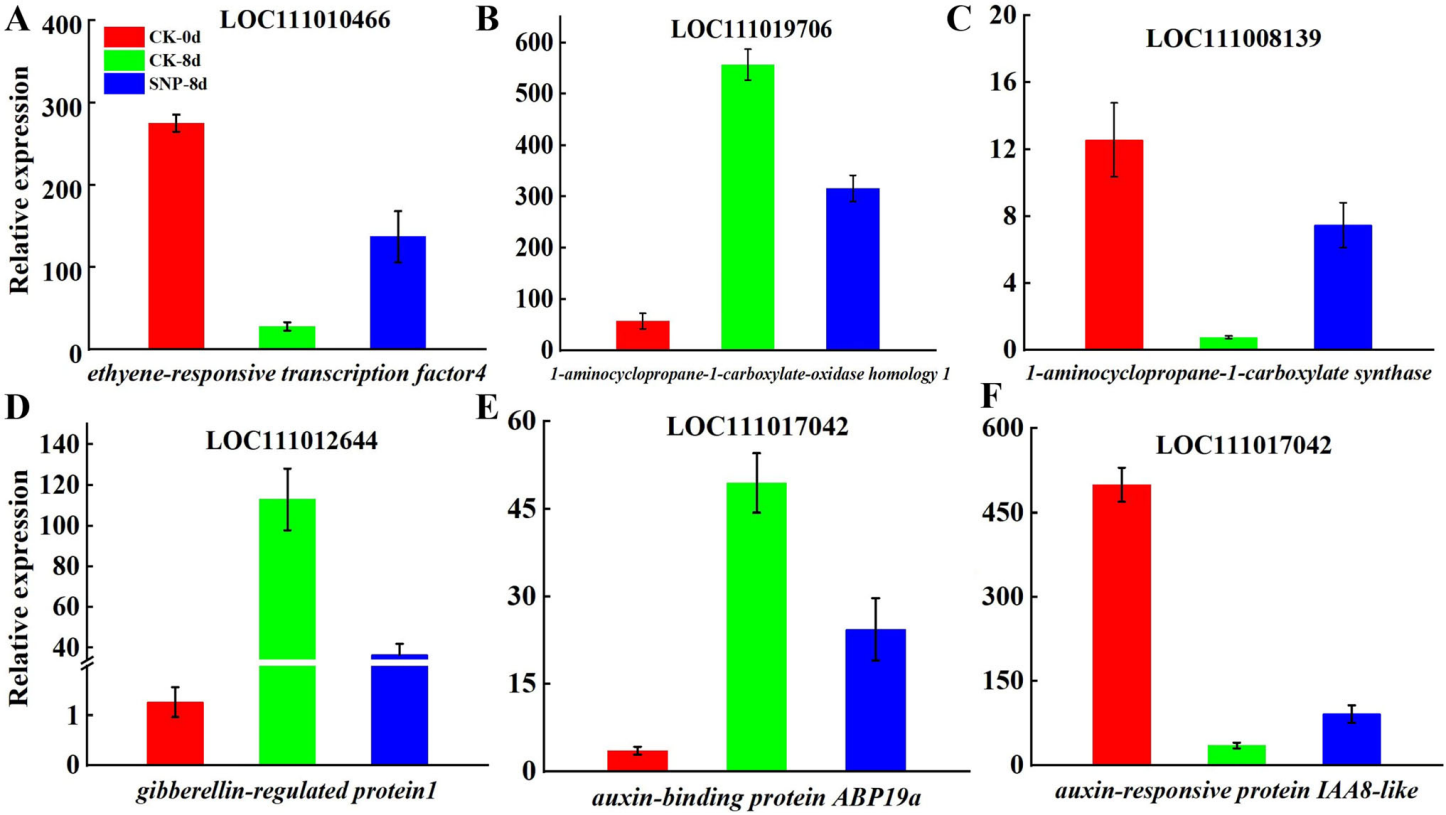
The mRNAs correlated with plant hormone synthesis and signal transduction in bitter melon fruit in the CK-0d group and the CK-8d and SNP-8d groups after eight days. **A** *Ethylene-responsive transcription factor 4* (*ERF4*). **B** *1-aminocyclopropane-1-carboxylate oxidase homolog 1-like* (*ACO1*). **C** *1-aminocyclopropane-1-carboxylate synthesis* (*ACS*). **D** *Gibberellin-regulated protein 1-like* (*GASA1*). **E** *Auxin-binding protein ABP19a* (*ABP19A*). **F** *Auxin response protein IAA8-like* (*IAA8*)

The results of a detailed analysis showed that the expression of *gibberellin-regulated protein 1-like* (*GASA1*), *ethylene responsive transcription factor 4* (*ERF4*), *1-aminocyclopropane-1-carboxylate oxidase homolog 1-like* (*ACO1*), *auxin-responsive protein SAUR 71-like* (*SAU71*), and *auxin-binding protein ABP19a* (*ABP19A*) was upregulated during natural ripening, while the expression of these genes was inhibited following SNP treatment (Fig. 3). We have identified genes related to plant hormones, including *ERF*, *abscisic acid receptor*, and *ACO*, which are independently regulated by SNP during the ripening and senescence process of bitter melon fruits. Interestingly, these genes were all positively regulated by SNP. These plant hormones-related DE mRNAs play an essential role in delaying the ripening and senescence of bitter melon after SNP treatment.

#### Genes involved in disease resistance of bitter melon fruit

In the CK-0d vs. CK-8d comparison group, 120 mRNAs were differentially expressed and were associated with genes involved in the plant–pathogen interaction pathway; the expression of 53 mRNAs increased, whereas that of 67 mRNAs decreased. For example, the expression of *serine/threonine-protein kinase PBL5* (*PBL5*)*, CDPK-related kinase 5-like* (*CRK5*)*, calcium-binding protein CML25* (*CML25*)*, 3-phosphoinositide-dependent protein kinase B-like isoform X1, calcium/calmodulin-regulated receptor-like kinase 1* (*CRLK1*), *serine/threonine-protein kinase PBS1* (*PBS1*), and *PBL5* was significantly upregulated. Moreover, the expression levels of the *serine/threonine-protein kinase PBL7* (*PBL7*), *WRKY transcription factor 50* (*WRKY50*), *serine/threonine-protein kinase-like protein ACR4* (*ACR4*)*,* and *receptor-like cytosolic serine/threonine-protein kinase RBK1* (*RBK1*) were analyzed, and most of these levels significantly decreased (Supplementary Appendix 4).

Some genes involved in plant defense and response to pathogens are also identified in bitter melon. In the CK-8d vs. SNP-8d comparison group, 16 differentially expressed mRNAs related to the plant–pathogen interaction pathway were identified. The expression of 14 of these mRNAs increased after SNP treatment compared to the control treatment, and the expression of 2 decreased. A detailed analysis of the expression patterns showed that the expression levels of *WRKY transcription factor 51* (*WRKY51*)*, calmodulin-like protein 11* (*CALM*)*, L-type lectin-domain containing receptor kinase IX.1-like* (*LRK91*)*, G-type lectin S-receptor-like serine/threonine-protein kinase B120* (*B120*)*, serine/threonine-protein kinase RIPK* (*RIPK*), and *protein EDS1L-like* (*EDS1L*) increased significantly after SNP treatment, while those of the *cysteine protease RD19A-like* (*RD19A*) and *receptor-like protein kinase THESEUS 1* (*THE1*) decreased (Supplementary Appendix 5). A comprehensive analysis of the above two comparison groups showed that the relative expression of the proteins *EDS1L-like* (*EDS1*), *B120*, and *RIPK* increased after SNP treatment and that the relative expression of *THE1* and *RD19A* decreased, while the opposite was observed for the CK-0d vs. CK-8d comparison group. Therefore, this may be related to the resistance of fruit during ripening (Table 2).

**Table 2:** Several differentially expressed mRNAs are related to disease resistance

ID	Nr annotation	Regulated	log2FC	
			CK-0d vs CK-8d	CK-8dvs SNP-8d
LOC111007015	*Protein EDS1L-like* (*EDS1L*)	Up	− 3.30	1.88
LOC111010444	*Receptor-like protein kinase THESEUS 1* (*THE1*)	Down	2.34	− 1.15
LOC111012488	*G-type lectin S-receptor-like serine/threonine-protein kinase B120* (*B120*)	Up	− 1.82	1.73
LOC111015466	*Cysteine protease RD19A-like* (*RD19A*)	Down	2.54	− 2.29
LOC111016464	*Serine/threonine-protein kinase RIPK* (*RIPK*)	Up	− 1.76	1.68

#### Genes involved in fruit color of bitter melon fruit

Based on transcriptome data, we did not find any key genes related to pigment accumulation in the carotenoid synthesis pathway. In the CK-0d vs. CK-8d group, in the porphyrin and chlorophyll metabolism pathway, many genes related to chlorophyll degradation were found to be upregulated, including *heme oxygenase 1, chloroplastic* (*AtHO1*), *chlorophyll(ide) b reductase NYC1, chloroplastic* (*NYC1*), *ferrochelatase-2, chloroplastic* (*HEMH*), *frataxin, mitochondrial* (*Fxn*), *magnesium-chelatase subunit Chl*H (CHLH), and *protein STAY-GREEN, chloroplastic-like* (*CaSGR*). In addition, the expression of flavonoid related genes increased, such as *shikimate O-hydroxycinnamoyltransferase* (*HCT*), *chalcone–flavonone isomerase* (*CHI*), *flavonol synthase/flavanone 3-hydroxylase* (*FLS1*), and *chalcone synthase-like* (*CHS*). After SNP treatment, the expression of chlorophyll metabolism regulated genes decreased, like *chlorophyll(ide) b reductase NYC1, chloroplastic* (*NYC1)*, and *heme oxygenase 1* (*AtHO1*); and the gene of *shikimate O-hydroxycinnamoyltransferase* (*HCT*) was increased in the flavonoid biosynthesis; the changes of these flavonoid synthase and chlorophyll metabolism mRNAs may be related to the changes in fruit color, because the yellow color of bitter melon fruit after 8 days is more significant than that after 0 days.

#### Genes involved in fruit flavor and aroma of bitter melon fruit

**Fig. 4:**
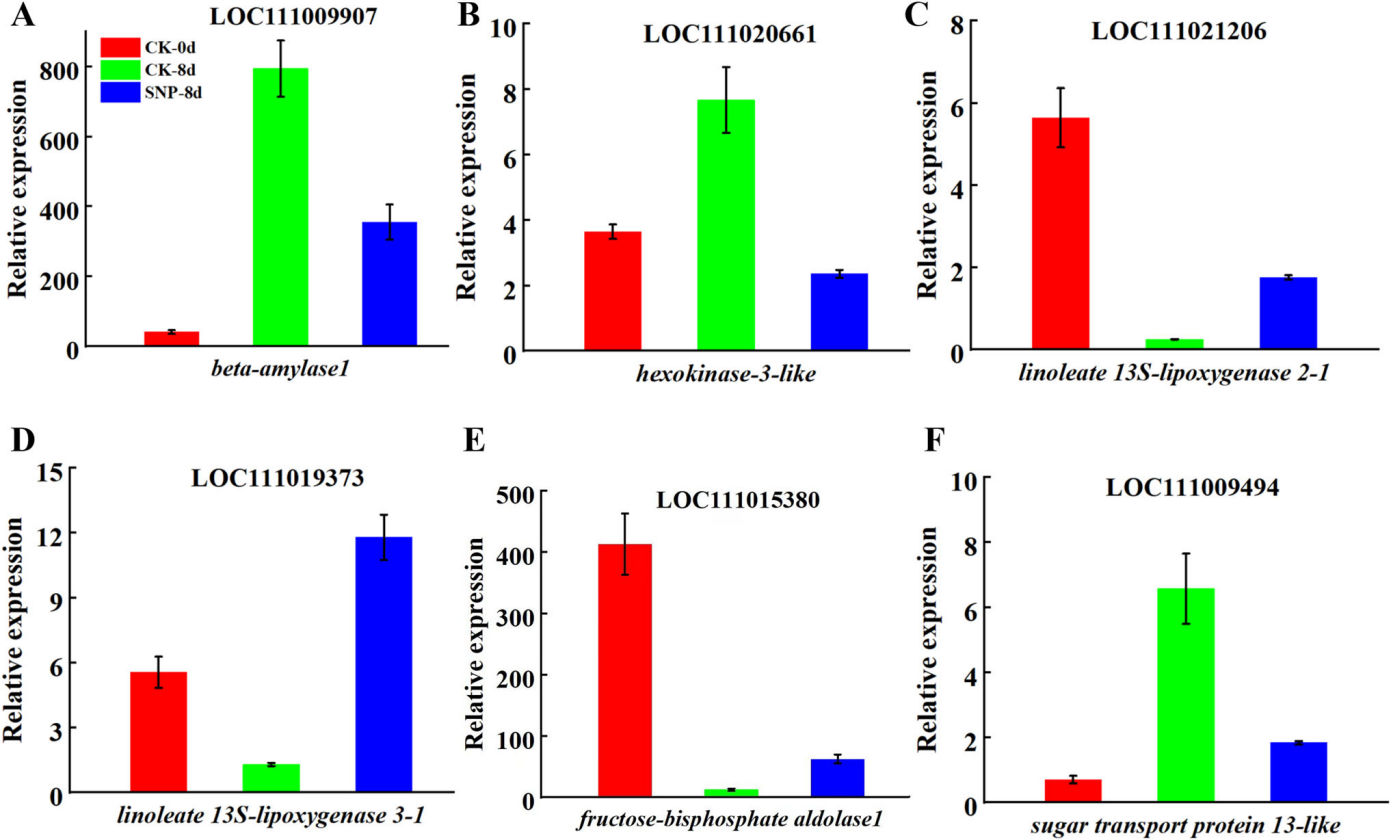
The mRNAs correlated with flavor and aroma in bitter melon fruit in the CK-0d group and the CK-8d and SNP-8d groups after 8 days. **A** *Beta-mylase 1* (*BAM1*). **B** *Hexakinase-3 like* (*HXK3*). **C** *Linoleate 13S-lipoxygenase 2-1* (*LOX21*). **D** *Linoleate 13S-lipoxygenase 3-1* (*LOX31*). **E** *Fructose-bisphosphate aldolase 1* (*FBA1*). **F** *Sugar transport protein 13 like* (*STP13*)

During the natural ripening stages (CK-0d vs. CK-8d), the results showed that there were seven genes for fruit flavor and aroma. Among them, *phenylalanine ammonia-lyase-like (PAL)*, *alcohol dehydrogenase-like 7* (*ADH7*), *fructokinase-like 2* (*FRK2*), *alpha-galactosidase 1-like* (*GAL1)*, and *linoleate 13S-lipoxygenase 2-1* (*LOX*) were downregulated. And the genes of *glutamate dehydrogenase 2* (*GDH2*) and *sucrose synthase 2* (*SUS2*) were upregulated. In addition, we identified a total of ten key genes that regulate the flavor of bitter melon fruit in the CK-0d vs. CK-8d and CK-8d vs. SNP-8d comparative groups. Among these genes, *beta-mylase 1* (*BAM1*) and *hexakinase-3 like* (*HXK3*), *sugar carrier protein A isoform X1*, and *sugar transport protein 13 like* (*STP13*), were upregulated in CK-0d vs CK-8d, but downregulated in CK-8d vs SNP-8d. And the genes of *phenylalanine ammonia-lyase-like* (*PAL*), *bidirectional sugar transporter SWEET16-like* (*SWT16*), *fructose-bisphosphate aldolase 1* (*FBA1*), *linoleate 13S-lipoxygenase 2-1* (*LOX21*), *linoleate 13S-lipoxygenase 3-1* (*LOX31*), and *pyruvate decarboxylase 1* (*PDC1*) were upregulated after SNP treatment. These DEGs mentioned above may be involved in the regulation of fruit flavor and aroma during the ripening process of bitter melon (Fig. 4).

### Effect of SNP treatment on the accumulation of metabolites in bitter melon fruit

**Fig. 5:**
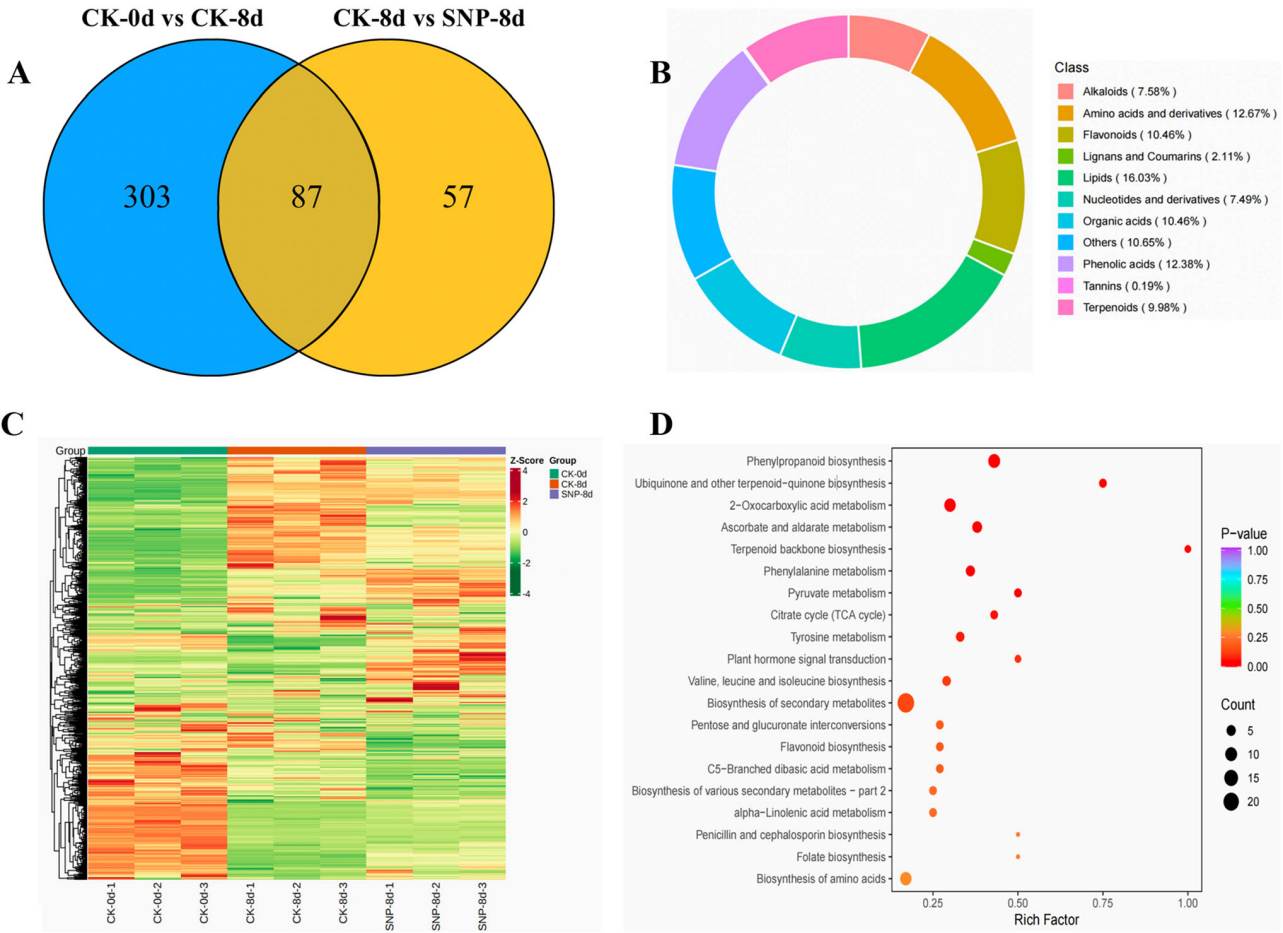
Impact of SNP treatment on metabolite levels in bitter melon fruit. **A** Venn diagram of different metabolites among the comparison groups; **B** classification of differentially accumulated metabolites that constitute the main components of bitter melon fruit in the CK-8d vs. SNP-8d comparison group; **C** heatmap of metabolites that differentially accumulated in the CK-0d, CK-8d and SNP-8d groups; **D** KEGG pathways of DEGs in the CK-8d vs. SNP-8d comparison group. (Each circle in the figure represents a comparison group, and the number of overlapping parts represents the common differentially abundant metabolites between the comparison groups.)

By comparing the metabolic data of bitter melon fruit in the CK-0d and CK-8d groups, we found that 390 metabolites differentially accumulated, of which 207 increased in abundance and 183 decreased in abundance (Fig. 5A). These differentially accumulated metabolites may represent the main components of bitter melon fruit and were divided into 11 categories. The largest proportion was lipids (16.03%), followed by amino acids and their derivatives (12.67%), phenolic acids (12.38%), organic acids (10.46%), terpenoids (9.98%), alkaloids (7.58%), nucleotides and their derivatives (7.49%), lignans and coumarins (2.11%), and tannins (0.19%) (Fig. 5B). Moreover, the differentially accumulated metabolites are mainly involved in pathways associated with linoleic acid metabolism, arginine biosynthesis, purine metabolism, fructose and mannose metabolism, histidine metabolism, ubiquitin, and terpenoid quinone metabolism (Supplementary Appendix 6).

By comparing the CK-8d and SNP-8d groups, we identified 144 differentially accumulated metabolites. KEGG pathway analysis showed that the main changes in response to SNP involved metabolites such as those that participate in phenylpropanoid biosynthesis, the biosynthesis of ubiquinone and other terpenoid-quinones, 2-oxo-carboxylic acid metabolism, ascorbate and aldarate metabolism, terpene backbone biosynthesis, and phenylalanine metabolism (Fig. 5C, D, Supplementary Appendix 7).

The relative contents of phenolic acids, lipids, terpenoids, and flavonoids in the two control groups were significantly different. In the CK-0d vs. CK-8d comparison group, coumarin-4-O-glucoside increased in abundance by 146,025-fold. Several free fatty acids, such as methyl linolenate, 15(R)-hydroxylinolic acid, 9-hydroperoxy-9Z,11E-octadecadienoic acid, (9Z,12Z)-(7S,8S)-dihydroxyoctadeca-9,12-dienoicacid, 9-hydroxy-12-oxo-10(E),15(Z)-octadecadienoic acid, 12,13-epoxy-9-octadecenoic acid, hydroxy ricinoleic acid, 9-hydroxy-10,12,15-octadecatrienoic acid, (9Z,11E)-13-oxooctadeca-9,11-dienoic acid, and 9-hydroxy-13-oxo-10-octadecenoic acid, increased in abundance. However, the lipid substances lysoPE (6:1*, 17:1*, 16:0, 14:0, 18:3*, 18:1*, 18:0, 20:3*) and lysoPC (12:0, 16:1*, 7:2, 15:0*, 16:0, 17:0, 18:4, 15:1) decreased in abundance. In the CK-8d vs. SNP-8d comparison group, kuguasaponin A, which is a terpenoid unique to bitter melon fruit, presented the maximum abundance FC. Phenolic acids, including 3-*O-p*-coumaroylshikimic acid, sinapinadehyde, cinnamic acid, *p*-coumaryl alcohol, sinapic acid, and α-hydroxycinnamic acid*, also increased in abundance. Similarly, the abundance of LysoPE (18:3, 18:4, 20:5, 20:3*, 20:2, 18:0) and LysoPC (20:3, 20:2) increased. Thus, the free fatty acids and lipid substances of bitter melon fruit were increased during untreatment storage, but were decreased after SNP treatment.

We also identified metabolic substances that were independently regulated by SNP during the ripening and senescence process of bitter melon fruits. Through the analysis of metabolic substances, SNP treatment mainly affected phenolic acids, lipids, amino acids, and their derivatives. Especially, it had a significant regulatory effect on lipid substances, such as 12-hydroxydodecanoic acid, 9-hydroperoxy-10E, 12, 15Z-octadecatrienoic acid, and jasmonoyl-l-Isoleucine.

Additionally, based on the data, the flavonoid metabolites in the two comparison groups (CK-0d vs. CK-8d and CK-8d vs. SNP-8d) were mainly periodictol-3′-*O*-glucoside, apiferol, dihydrokaempferol-7-*O*-glucoside, and luteolin-7-*O*-glucuronide. These metabolites participate in the flavonoid synthesis pathway.

### Combined transcriptomic and metabolomic analysis

**Fig. 6:**
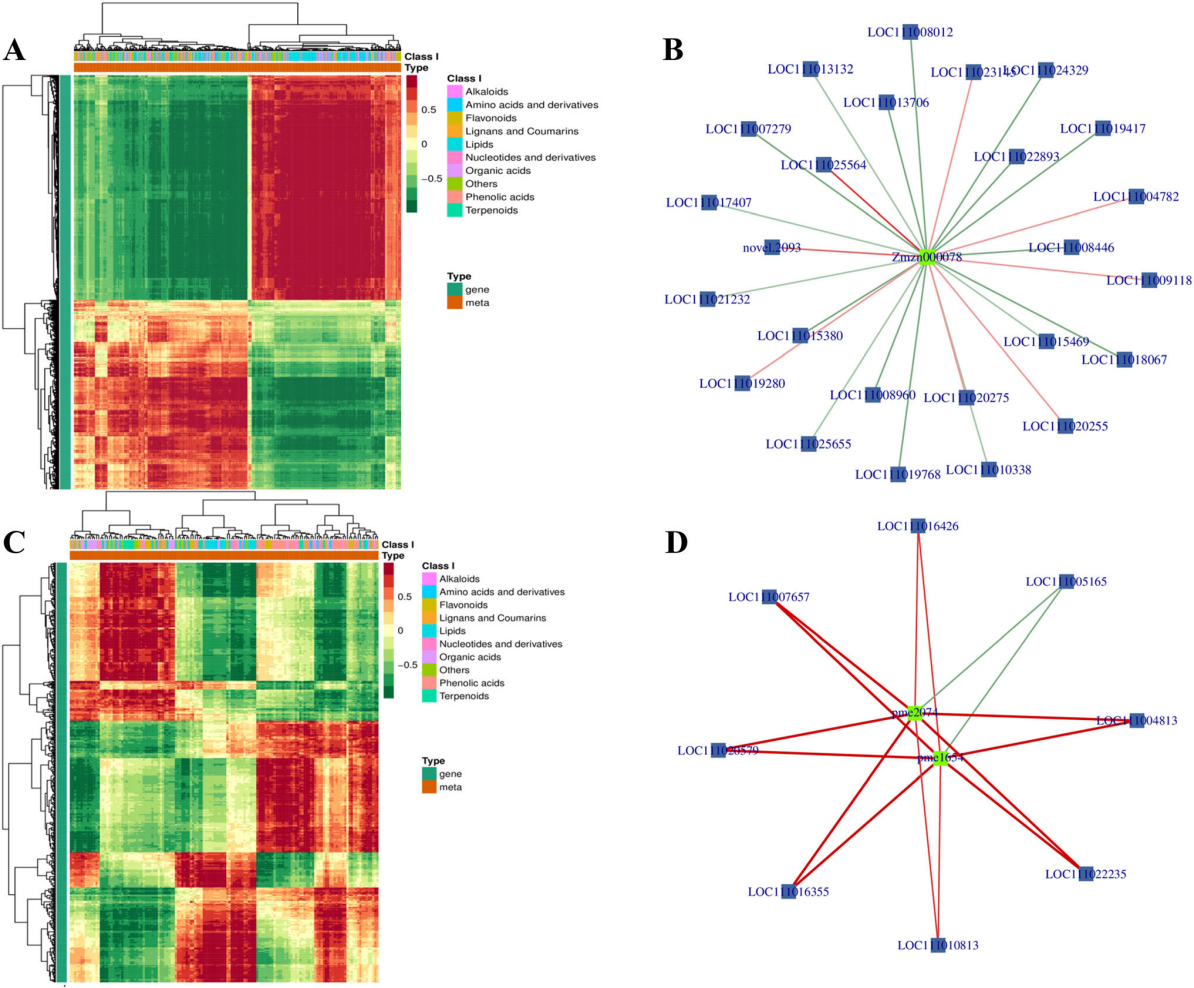
**A** Correlation clustering heatmap corresponding to the CK-0d vs. CK-8d comparison group. **B** Correlation network diagram of genes and dihydroxyacetone phosphate in the CK-0d vs. CK-8d comparison group. **C** Correlation clustering heatmap corresponding to the CK-8d vs. SNP-8d comparison group. **D** Correlation network diagram of genes and jasmonic acid in the CK-8d vs. CK-8d comparison group. (In the figure, metabolites are represented by green squares, genes are represented by blue squares, red lines represent positive correlations, and green lines represent negative correlations.)

Compared with independent analyses, a combined metabolomic and transcriptomic analysis can more specifically illustrate the relationship between DEGs and differentially abundant metabolites, the results of which are beneficial for clarifying the effects of SNP treatment on the ripening and senescence of bitter melon fruit. Correlation analysis was performed on the DEGs and differentially accumulated metabolites in the CK-0d vs. CK-8d and CK-8d vs. SNP-8d comparison groups (Fig. 6A, C).

In the CK-0d and CK-8d comparison, dihydroxyacetone phosphate was positively correlated to *aldehyde dehydrogenase family 2 member B7,* (*ALDH2b*), *2,3-bisphosphoglycerate-independent phosphoglycerate mutase* (*BPG-independent PGAM*), *leghemoglobin reductase* (*FLBR*), *alcohol dehydrogenase-like 7* (*ADHL7*), and *pyruvate dehydrogenase E1 component subunit alpha-1* (*PDHE1-A*) in the glycolysis/glycogenesis pathway (Fig. 6B, Appendix 8). Luteolin-7-*O*-glucoside (cynaroside) and kaempferol-3*-O*-glucoside (astragalin) were negatively correlated to *UDP-glycosyltransferase 83A1-like* (*UGT83A1*) and *UDP-rhamnose:rhamnosyltransferase 1* (*FaRT1*) in the flavone and flavonol biosynthesis pathways.

**Fig. 7:**
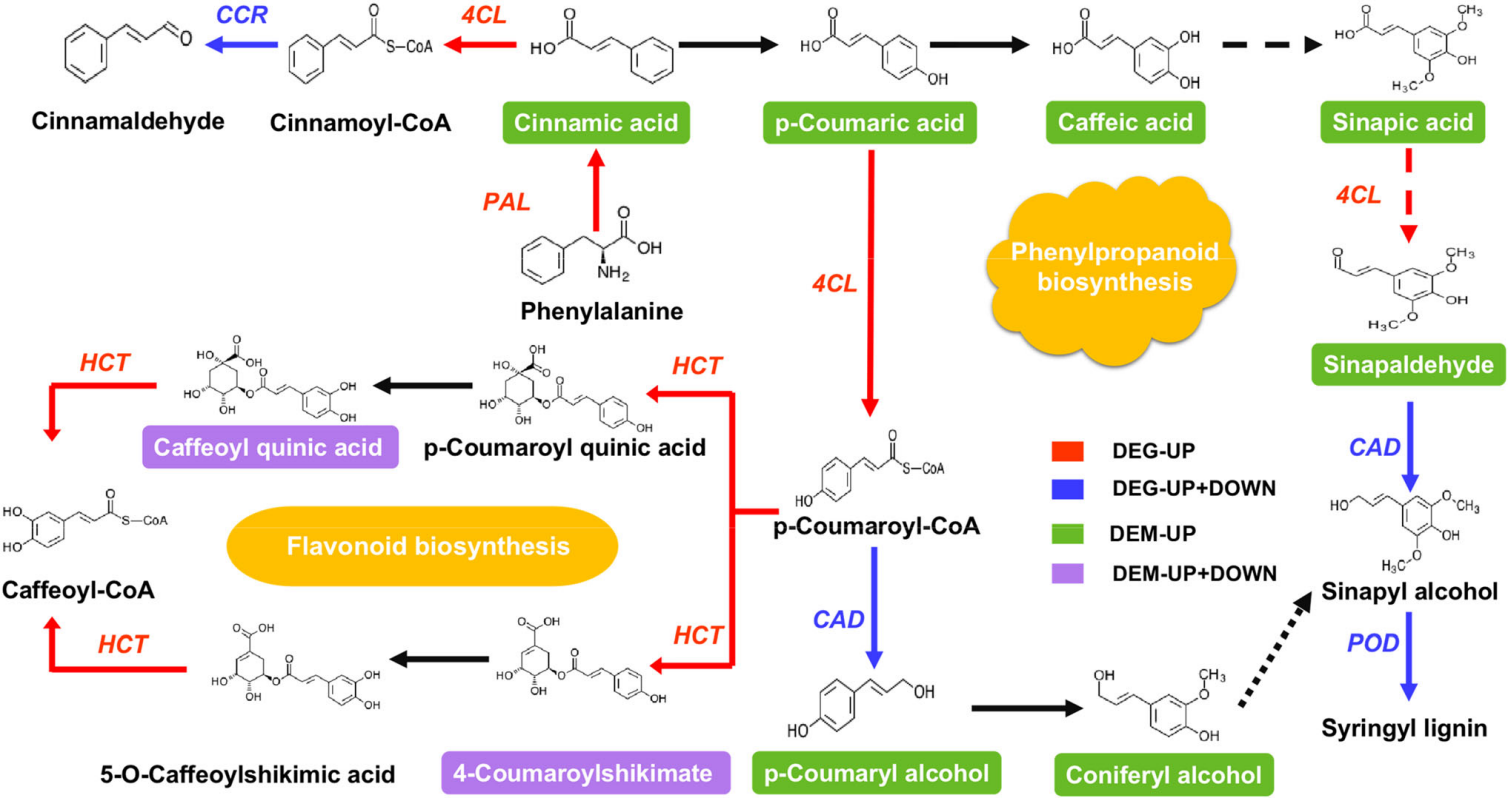
Interactions between related DEGs and differentially accumulated metabolites in the phenylpropanoid biosynthesis pathway and flavonoid biosynthesis pathway at the same time in the CK-8d vs. SNP-8d comparison group. Green boxes represent an increase, purple boxes represent up and down in differential accumulation, while red and green boxes represent changes of DEGs

In the CK-8d vs. SNP-8d comparison group, in the plant hormone signal transduction pathway, the differentially accumulated metabolites jasmonic acid and jasmonoyl-L-isoleucine were negatively correlated to cysteine-rich receptor-like protein kinase 2 (CRK2), while the auxin-responsive protein IAA16-like (IAA16) and auxin-responsive protein IAA8-like (IAA8) were positively correlated (Fig. 6D, Appendix 9). In addition, the differentially abundant metabolites *p*-coumaryl alcohol, sinapinaldehyde, cinnamic acid, and *p*-coumaric acid were positively correlated to *phenylalanine ammonia-lyase* (*PAL*), *4CL*, *POD*, *cinnamyl alcohol dehydrogenase* (*CAD*), and *cinnamyl coenzyme A reductase* (*CCR*) in the phenylpropanoid biosynthesis KEGG pathway. At the same time, after SNP treatment in the flavonoid biosynthesis pathway, it had a positive regulation on *HCT*, and then, the different metabolites caffeioyl quinic acid and 4-coumaroylshikimate were significantly changed, thus affecting the color change in bitter melon fruit (Fig. 7).

### Effect of SNP treatment on the changes in protein abundance in bitter melon fruit

Proteomic analysis of the fruit in CK-0d vs. CK-8d comparison group revealed a total of 1603 DEPs, among which 863 increased in abundance and 740 decreased in abundance. The results of a KEGG pathway enrichment analysis showed that the genes encoding the DEPs were primarily enriched in phenylpropanoid biosynthesis; galactose metabolism; valine, leucine, and isoleucine degradation; fatty acid degradation; ABC transporters and flavonoid biosynthesis (Supplementary Appendix 10). According to the results of a proteomic analysis of the fruit in the CK-8d vs. SNP-8d comparison group, we revealed a total of 306 DEPs, among which 175 increased in abundance and 131 decreased in abundance. And 306 DEPs were found to be involved primarily in plant hormone signal transduction, phenylpropanoid biosynthesis, glutathione metabolism, carotenoid biosynthesis, and starch and sucrose metabolism (Supplementary Appendix 11).

By screening and analyzing the fruit in the CK-8d and SNP-8d groups, we found many different proteins that may be regulated by SNP were found during the maturation and senescence of bitter melon fruit. Through a detailed analysis of the CK-8d vs. SNP-8d comparison group data, we identified several differentially abundant proteins (DAPs), that are strongly active in bitter melon fruit, such as PG, pectate lyase (PL), and PME; DAPs that are related to plant hormone syntheses and signal transduction, such as PYL9, the transcription factor MYC2-like (MYC2), and jasmonic acid-amido synthetase (JAR1); and DAPs that are involved in disease resistance, such as lysM domain receptor-like kinase 3 (LYK3) and the putative leucine-rich repeat receptor-like protein kinase At2g19210. Although no crucial gene changes were found in the carotenoid synthesis pathway, the respective protein changes were significant. The differentially expressed proteins involved in fruit color, such as zeta-carotene desaturase (ZDS), NADPH-dependent aldehyde reductase 1, chloroplastic-like, lycopene beta cyclase (LCYB), prolycopene isomerase (CRTISO), and secoisolariciresinol dehydrogenase-like (SDH).

By combining the CK-0d vs. CK-8d and CK-8d vs. SNP-8d comparison group data, we identified 65 DEPs regulated by SNP during the ripening and senescence of bitter melon fruit. Detailed analysis of their expression patterns showed that the jasmonic acid-amido synthetase (JAR1) and carboxylesterase 2 (CES2) which in plant hormone synthesis and signal transduction pathway were increased, as well as lysM domain receptor-like kinase 3 (LYK3) involved in plant–pathogen interaction. And in the carotenoid synthesis pathway, the protein zeta-carotene desaturase (ZDS), NADPH-dependent aldehyde reductase 1, chloroplastic-like, lycopene beta cyclase (LCYB), and prolycopene isomerase (CRTISO) were increased in abundance following SNP treatment. However, in the phenylpropanoid biosynthesis and galactose metabolism, peroxidase (POD) and galactinol-sucrose galactosyltransferase (RFS) proteins decreased in abundance (Table 3, Supplementary Appendix 12).

**Table 3:** Relative expression levels of select proteins in bitter melon fruit during ripening and senescence

Proteins	Regulated	log2FC	
		CK-0d vs CK-8d	CK-8dvs SNP-8d
LysM domain receptor-like kinase 3 (LYK3)	Up	− 1.06	0.75
Carboxylesterase 2 (CES2)	Up	− 1.13	0.79
Pectate lyase (PL)	Up	− 1.77	0.95
Jasmonic acid-amido synthetase (JAR1)	Up	− 0.627	0.747
Peroxidase (POD)	Down	2.72	− 0.72
Galactinol-sucrose galactosyltransferase (RFS)	Down	2.44	− 0.92
Zeta-carotene desaturase (ZDS)	Down	1.48	− 0.69
NADPH-dependent aldehyde reductase 1	Down	8.04	− 1.42
Lycopene beta cyclase, chloroplastic/chromoplastic (LCYB)	Down	2.02	− 0.75
Prolycopene isomerase (CRTISO)	Up	− 1.62	0.87

### Combined transcriptomic and proteome analysis

In the CK-0d vs. CK-8d comparison group, genes and the respective proteins are mainly enriched in phenylpropanoid biosynthesis, cysteine and methionine metabolism, peroxisome, fatty acid metabolism, and galactose metabolism. Some genes and proteins have similar and different change trends. Among the phenylpropanoid biosynthesis, the genes *4CL*, *CAD*, and *CCR* and their respective proteins were have similar trends. In comparing CK-8d vs SNP-8d, genes and the respective proteins are mainly enriched in phenylpropanoid biosynthesis, MAPK signaling pathway—plant, plant hormone signal transduction, and starch and sucrose metabolism. The genes and proteins related to phenylpropanoid biosynthesis have different change trends, like shikimate O-hydroxycinnamoyltransferase (HCT). The genes and proteins which indicated the same similar trends involved the plant hormone signal transduction. The case in point is that the PYL and transcription factor MYC2 increased, which may have a promotional effect on proteins.

## Discussion

Bitter melon (*Momordica charantia* L*.*) is a gourd vegetable species native to tropical Asia. However, bitter melon fruits are very sensitive to temperature. Indeed, when stored at room temperature, they are very prone to yellowing, softening, aging, and decaying, which limit their shelf-life (Lin et al. 2020). Application of NO is a regulatory technique that is widely used for the postharvest preservation of fruit and vegetables to delay ripening and senescence (Madebo et al. 2022). At present, many studies have shown that NO can delay the ripening of grape, peach, and winter jujube fruit via its antagonistic effect on ethylene. Thus, it is able to improve their stress resistance during storage, maintain their postharvest quality, and prolong their shelf-life (Zhang et al. 2019; Han et al. 2018; Zhao et al. 2020). In our study, bitter melon fruit treated with NO showed milder symptoms of ripening and senescence compared with those of untreated bitter melon fruit. These results indicated that NO could delay the ripening and senescence process of bitter melon fruit.

The texture of fleshy fruit is an important quality feature of mature fruit. Softening is an irreversible phenomenon that occurs in most fleshy fruit during ripening and is not conducive to consumer preference for sensory and taste of fruit (Gao et al. 2020; Shi et al. 2022; Tucker et al. 2017). Fruit softening is mainly regulated by the expression of genes encoding cell wall-modifying enzymes, such as β-galactosidase (β-Gal), PL, PME, pectin acetylesterase (PAE), rhamnose lactose lyase (RGL), cellulose synthase (CSLE), PGA, and pectin esterase (PE). These genes interact with each other to participate in the synthesis and degradation of cell walls (Sun et al. 2015; Araque et al. 2019; Wang and Seymour 2022). In our study, following NO treatment (CK-8d vs. SNP-8d), the expression levels of several important genes participating in cell wall modification, such as *β-Gal8, EXLA2, PGL3*, and *polygalacturonase 5* (*PGLR5*)*,* were inhibited, while the expression of *cellulose synthase-like protein E6* (*CSLE6*)*, CSLE1,* pectinesterases (*PE1, PE2*), and *endochitinase a* (*CHIX*), increased (Table 1). Studies have also indicated that an increase in PG activity can accelerate the softening of fruit and vegetables (Fan et al. 2019; Zhao et al. 2019). We found that NO treatment inhibited the expression of *β-Gal8*, *EXLA2*, and *PGLR5*, which is consistent with the delayed softening of green pepper fruit (Ma et al. 2021). Therefore, our results suggest that NO treatment can affect the expression of genes related to cell wall degradation, thus delaying the ripening and senescence of bitter melon fruit.

Plant hormones are important signaling compounds and play important roles throughout the whole process of fruit growth, development, and maturity. Our research revealed several genes related to plant hormone synthesis and signal transduction, including abscisic acid (ABA), auxin (IAA), and gibberellin regulatory proteins as well as jasmonic acid transduction factors (Bai et al. 2021). In most cases, Aux/IAAs are the main signaling components in the auxin signaling pathway, and these hormones can inhibit fruit ripening via their antagonistic role(s) to ethylene or ABA (Liao et al. 2018; Sravankumar et al. 2018). Moreover, we identified transcription factors that are involved in ethylene biosynthesis-, sensing-, and signaling-related genes in bitter melon fruit. However, their roles in the storage of bitter melon fruit need to be further clarified. In addition, we found that in the control group, the expression levels of *GASA1*, *ERF4*, *ACO1*, *SAU71*, and *ABP19A* increased, but after SNP treatment, their levels decreased. Ethylene response factors play important roles in the ethylene signaling pathway and can regulate the expression of downstream genes in response to fruit ripening (He et al. 2020). Research has shown fruits with overexpression of *LeERF1* in tomatoes exhibit an ethylene reaction that accelerates the ripening and softening of tomato fruits, while when *LeERF1* expression is inhibited, tomato fruit ripening slows down (Li et al. 2007). Therefore, in this study, the decreased expression of *ERF4* may delay the ripening and senescence of bitter melon fruits. Taken together, these results suggest that the above-mentioned genes may play essential roles in regulating the reduction in ethylene biosynthesis-related enzymes and signal transduction-related transcripts after SNP treatment during fruit storage.

Phenylpropanoid metabolism is one of the most important metabolic pathways in plants and gives rise to metabolites, including lignin, flavonoids, lignans, phenylpropanoid esters, hydroxycinnamic acid amides, and sporopollenin (Dong and Lin 2021). The four key enzymes that affect lignin cell wall deposition are *PAL, CAD, POD,* and *4CL* (Zhou et al. 2018; Vanholme et al. 2019; Barros et al. 2015). In the present study, we found that SNP treatment increased the transcription of *PAL*, while the activity of *CAD*, *4CL CAD,* and *POD* both increased and decreased, which collectively lead to the accumulation of *p*-coumaryl alcohol, sinapinaldehyde, cinnamic acid, and *p*-coumaric acid. These results are similar to previous results reported in navel orange and cherry tomato fruit, in which SNP treatment was shown to increase the transcript level of *PAL* and *C4H* and promote lignin accumulation in the fruit (Yang et al. 2021; Li et al. 2019). It was also found that NO treatment activated *PAL*, *C4H*, and *4CL* transcription in postharvest muskmelon fruit infected with black spot, suggesting that NO can promote lignin biosynthesis by increasing the activity of the phenylpropanoid pathway, thereby improving the resistance of muskmelon fruit to black spot (Yan et al. 2019). Indeed, the increase in lignin content may be due to NO treatment, which not only activated the phenylpropanoid pathway but also enhanced *POD* activity.

In addition, changes in the expression of genes associated with disease resistance were detected. The mRNAs encoding *CALM, LRK91, B120, RIPK*, and *EDS1L* increased significantly after SNP treatment. Thus, we speculate that SNP treatment could enhance the disease resistance of bitter melon fruit and thereby extend their shelf-life.

**Fig. 8:**
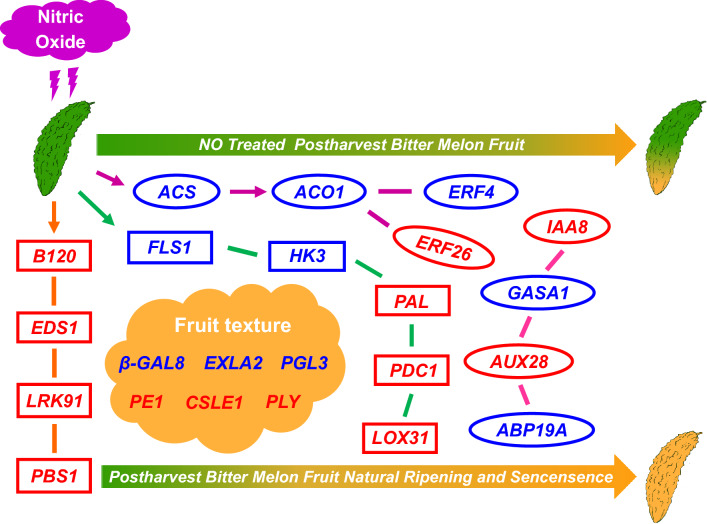
Model of the regulatory mechanism through which NO treatment delays the maturation and senescence of bitter melon fruit. The red color represents gene upregulation, and the blue color represents gene downregulation

A regulatory model has been developed to describe the possible regulatory mechanism of NO treatment in delaying the maturation and senescence of bitter melon fruit (Fig. 8). It mainly regulates genes related to fruit texture, aroma, plant hormone signal transduction, and disease resistance.

## Conclusion

The purpose of this study was to investigate the changes in the molecular physiology of bitter melon fruit during postharvest storage following NO treatment. Our results revealed that NO treatment could delay the physiological changes and maintain the sensory qualities and firmness of bitter melon fruit during postharvest storage. Transcriptomic analysis showed that NO treatment significantly affected the key genes related to textural changes, flavor and aroma production and plant hormone synthesis, perception, and signal transduction. Our metabonomic analysis showed that the differentially abundant metabolites were mainly phenolic acids, terpenoids, flavonoids, and lipids. When our transcriptomic and metabolomic data were combined, it was found that the changes in the expression of the *PAL, CAD, 4CL,* and *POD* genes, which are related to phenylalanine synthesis, may promote the accumulation of coumarin alcohol, octanal, cinnamic acid, and *p*-coumarinic acid following NO treatment. The combined analysis of transcriptome and proteomics revealed differentially expressed proteins related to phenylpropane biosynthesis and plant hormone signal transduction. Furthermore, NO treatment increased and decreased the expression of various mRNAs, revealing the regulatory process that delayed the maturation and senescence of bitter melon fruit.

## Materials and methods

### Fruit treatment

Bitter melon fruits were purchased from the Beijing Haidian Guo Shu Hao supermarket. Fruits that were of the same size and color, free of deformities, not overripe and without cracks were selected and returned to the laboratory within 2 h. The bitter melon fruits were divided into two groups (100 fruits in each group): the fruit in one group was soaked in a 0.25 mmol/L NO donor [sodium nitroprusside (SNP)] solution for 20 min; the fruit in the other (control) group was immersed in deionized water for 20 min. After drying naturally in a cool place, the fruit samples were placed in a 0.03 mm polyethylene plastic bag, which was folded and sealed, and then stored at 20 °C (humidity 85–90%) for 8 days. Fruit sensory attributes, firmness, and quality were measured every 2 days. For the sampling of bitter melon fruits, they were cut into cubes from the middle of equator (excluding all seeds and capsules), frozen in liquid nitrogen, and stored at -80 °C for multi-omics analysis. Three physiological experiments were repeated, with three bitter melon fruit constituting each group. Each experimental group included three biological replicates.

### Sensory evaluation and firmness

An evaluation team, comprised of ten group members who had received standard training, was recruited. A nine-point system (Table 4) was adopted to evaluate the quality of the two groups of bitter melon fruit, on the basis of the hardness, color, luster, and rotten area. The higher the sensory score, the better the quality of the bitter melon fruit was, and the maximum evaluation score was nine points.

**Table 4:** Nine-point scale represents the quality of bitter melon fruit from good (9) to bad (1)

Score	Hardness	Color and luster	Decay degree
9	High hardness, no wilting	Bright green color	No decay
8–7	Firm and slightly wrinkled	Green slightly yellowish, slightly darker	Rotten area < 1/5
6–5	Soft and wrinkled on the surface	Yellowish green, increased yellow area, dull color	Rotten area < 1/4
4–3	Soft and woolly, with deep folds	Brownish yellow, no color	Rotten area < 1/3
2–1	Wilted	Yellow	Decayed area > 1/2

A 4 mm GY-J digital force meter probe was used to determine the tissue strength (expressed in N) at three locations along the equatorial region of the center of the bitter melon fruit.

### Transcriptomic analysis

Staff at Metware Biotechnology Co., Ltd. (Wuhan, China) generated a cDNA library via the Illumina sequencing platform. Sequencing quality control (basic quality value and basic content distribution among the sequence data) was performed, and clean data were obtained (The reference genome used in this project is gcf_00995035.1_asm199503v1_genomic.fNa.gz., available at ftp://ftp.ncbi.nlm.nih.gov/genomes/all/GCF/001/995/035/GCF_001995035.1_ASM199503v1/). The genome structure annotation file used is gcf_00995035.1_asm199503v1_Genomic.gff.gz. The sequences of the clean reads after quality control were compared with the reference genome sequence via HISAT2 (Subramanian et al. 2005; Kim et al. 2015) to determine the location of the reference genes and identified genes, as well as the specific sequence characteristics of the sequenced samples. StringTie (Pertea et al. 2015) was used to extract new gene sequences from the genome, and Diamond (Buchfink et al. 2015) was used to compare the new gene sequences with the information housed in the Kyoto Encyclopedia of Genes and Genomes (KEGG), Gene Ontology (GO), nonredundant (Nr), and EuKaryotic Orthologous Groups (KOG) databases to obtain annotation information. The functions of these genes were predicted by KEGG function enrichment, and the databases used refer to previous studies (Fu et al. 2022). The information from two databases, the PlnTFDB (Pérez-Rodríguez et al. 2010) and PlantTFDB (Jin et al. 2016), were integrated using the software iTAK (Zheng et al. 2016). To ensure that the number of fragments truly reflected the expression level of transcripts, it was necessary to normalize the number of mapped reads and the length of transcripts in the sample [fragments per kilobase of transcript per million mapped reads (FPKM)] (Trapnell et al. 2010). For samples with biological replicates, DESeq2 (Love et al. 2014; Varet et al. 2016) was used to conduct differential expression analysis among sample groups to obtain sets of differentially expressed genes (DEGs) between two biological conditions [filter conditions: | log2(fold-change [FC]) |≥ 1 and a false discovery rate (FDR) < 0.05].

### 4D-DIA proteomic analysis

The fruit samples were separated via a NanoElute system with a nanoliter flow rate. The mixed samples were separated via chromatography and then evaluated by a timsTOF Pro mass spectrometer operating in ddaPASEF mode. DIA-NN (v1.8.1) software was used to quantify the proteins, and the MaxLFQ intensity of each protein in the different samples was determined from the search results. Then, the relative quantitative values (Rs) of the proteins in the different samples were obtained by standardized processing through centralized transformation. The calculation formula used is as follows: *R*_*ij*_ = *I*_ij_/Median(*I*_j_), where *I* represent the sample and *j* represents the protein. Ratios of the expression of the samples were calculated for subsequent analysis. After standardized treatments were applied, a quantitative analysis of the proteins was performed to identify differentially expressed proteins (DEPs) (the screening criteria included a significance of *P* < 0.05 and a quantitative expression FC > 1.50). The DEPs in each comparison group were analyzed, and four different types of enrichment analysis of the DEPs in each comparison group were carried out: GO classification, KOG functional classification, KEGG pathway enrichment classification, and protein domain classification to understand the functional characteristics of the different proteins.

### Metabolomic analysis

Qualitative and quantitative analysis of metabolites was performed mainly on the basis of the self-constructed database MetWare database (MWDB). The qualitative analysis of the metabolites was carried out according to the second-order spectral information, and the software Analyst 1.6.3 was used to process the mass spectral data. Principal component analysis (PCA) was subsequently performed on samples (including the quality control samples) to analyze whether there were differences among groups (Chen et al. 2009). Orthogonal partial least-squares discriminant analysis (OPLS-DA), which combines orthogonal signal correction (OSC) and PLS-DA and uses partial least-squares discriminant analysis (PLS-DA), was also performed. R software was used to analyze the irrelevant differences (Chong and Xia 2018; Thévenot et al. 2015) to identify the different variables. At the same time, the differentially accumulated metabolites were further screened on the basis of their *P* value according to the results of univariate analysis.

### Combined transcriptome and metabolomic analysis

Metabolites can reflect the activity of metabolic pathways. Compared with independent analysis, a combined analysis of metabolomics and transcriptomics allows a better and more comprehensive interpretation of the regulatory mechanisms underlying the transcription of genes involved in metabolic pathways (Jozefczuk et al. 2010). According to the combined analysis of the results of the differentially accumulated metabolites and DEGs, the same group of DEGs and differentially accumulated metabolites were enriched in the same KEGG pathways. The expression and accumulation values of the genes and metabolites, respectively, in the samples with Pearson correlation coefficients between gene expression levels and metabolite accumulation being calculated using the COR function. To determine significance, the correlation coefficient criterion was set at greater than 0.80, and the *P* value as less than 0.05.

### Supplementary Information

Below is the link to the electronic supplementary material.

Supplementary file1 (XLSX 3404 KB)

## Data Availability

The datasets generated and/or analyzed during the current study are available from the corresponding author on request.
